# Optimization of left ventricular ejection fraction measurement by two-dimensional echocardiography in patients with repaired tetralogy of Fallot: comparison of geometric methods with cardiovascular magnetic resonance

**DOI:** 10.1186/1532-429X-14-S1-P108

**Published:** 2012-02-01

**Authors:** Jimmy C Lu, Gregory J Ensing, Sunkyung Yu, Thor Thorsson, Janet E Donohue, Adam L Dorfman

**Affiliations:** 1 Pediatrics and Communicable Diseases, Division of Pediatric Cardiology, University of Michigan, Ann Arbor, MI, USA

## Background

In patients with repaired tetralogy of Fallot (rTOF), left ventricular ejection fraction (LVEF) predicts adverse clinical outcomes. Cardiovascular magnetic resonance (CMR) is the gold standard for LVEF measurement, but two-dimensional echocardiography (2DE) is commonly used for serial evaluation of LVEF. The optimal 2DE method for LVEF measurement and limiting factors in this population are not known.

## Methods

This single-center retrospective study included all patients with rTOF with CMR performed 2007-2010 without general anesthesia and 2DE within 3 months of CMR, with adequate images for analysis by all 2DE methods. Two investigators blinded to CMR results measured LVEF from 2DE studies by biplane Simpson’s (BiS) method (using apical 4-chamber and apical or parasternal 3-chamber images), 5/6 area*length (AL), and visual estimate. Two investigators blinded to 2DE results measured LVEF from CMR by Simpson’s method, as well as by AL, to test validity of geometric assumptions. An investigator re-evaluated each modality at least one month later.

## Results

In 20 patients (28.5±14.7 years old, 40% female), visual estimation by 2DE best approximated LVEF by CMR (table), but with high interobserver variability (median 14.8%). LVEF by AL correlated moderately with CMR, but with higher intraobserver (median 7.1% vs. 2.9%, p=0.004) and interobserver variability (median 11.1% vs. 3.8%, p=0.004) than CMR; LVEF by BiS correlated poorly with CMR. AL method on CMR closely agreed with Simpson’s method on CMR. Relative to CMR, 2DE underestimated both short-axis area (diastolic 19.6±6.0 vs. 25.2±6.9 cm2, p=0.01; systolic 9.8±3.4 vs. 13.3±4.9 cm2, p=0.01) and LV length (diastolic 7.4±0.7 vs. 8.8±1.0 cm, p<0.0001; systolic 6.3±0.9 vs. 7.5±1.0 cm, p=0.0001). AL method did not improve with use of 3-chamber length. Substituting CMR short-axis area improved correlation (r=0.80, p<0.0001) more than substituting CMR LV length (r=0.70, p=0.001). Intraobserver and interobserver variability of 2DE systolic short-axis area were higher in systole (median 12.7% and 15.4%) than in diastole (median 4.0% and 10.2%).

**Table 1:** Correlation and agreement of 2DE methods with CMR

Method	Mean LVEF (SD)	Correlation	Mean difference (limits of agreement)
CMR (Simpson's)	53.3 (7.2)	-	-
CMR AL	55.7 (8.9)	r=0.90, p<0.0001	-2.5 (-10.1 to 5.2)
2DE visual estimate	56.8 (10.0)	r=0.69, p=0.001	-3.5 (-17.8 to 10.8)
2DE AL	57.6 (8.1)	r=0.59, p=0.01	-4.3 (-18.1 to 9.4)
2DE BiS	57.9 (9.5)	r=0.35, p=0.13	-4.6 (-23.7 to 14.4)

## Conclusions

In adults with rTOF, AL method better correlates with CMR than BiS, but with high intra- and interobserver variability for all 2DE methods. Lack of agreement is affected predominantly by 2DE-derived areas, particularly systolic, rather than ventricular length or geometric assumptions. Strategies to optimize image position and border detection are most likely to improve 2DE performance in this population.

## Funding

None.

